# Association between Fasting Ketonuria and Advanced Liver Fibrosis in Non-Alcoholic Fatty Liver Disease Patients without Prediabetes and Diabetes Mellitus

**DOI:** 10.3390/nu13103400

**Published:** 2021-09-27

**Authors:** Kiyoung Lim, Minkyu Kang, Junggil Park

**Affiliations:** Department of Internal Medicine, College of Medicine, Yeungnam University, Daegu 42415, Korea; lky8731@naver.com (K.L.); gsnrs@naver.com (J.P.)

**Keywords:** diabetes mellitus, ketone bodies, nonalcoholic fatty liver disease, fibrosis

## Abstract

Ketone body production, an alternative fuel upon low glucose availability, reduces hepatic fat accumulation. However, its clinical implications have not been established in patients with nonalcoholic fatty liver disease (NAFLD). We investigated the association between spontaneous fasting ketonuria and liver fibrosis in patients with NAFLD without prediabetes and diabetes mellitus (DM). A total of 6202 patients with ultrasound confirmed NAFLD without prediabetes and DM were enrolled in the study. Using low cut off values of NAFLD fibrosis score (NFS) and fibrosis-4, liver fibrosis was defined as an intermediate–high probability of advanced liver fibrosis. Of the 6202 NAFLD patients, 360 (5.8%) had ketonuria. Compared to the patients without ketonuria, patients with ketonuria were younger (41.1 vs. 44.6 years, *p* < 0.001), had lower levels of glucose (87.2 vs. 91.0 mg/dL, *p* < 0.001), and homeostatic model assessment for insulin resistance (1.0 vs. 1.5, *p* < 0.001). The presence of ketonuria had an inverse association with liver fibrosis, assessed using both NFS (final adjusted odds ratio [aOR], 0.67; 95% confidence interval [CI], 0.45–1.01) and fibrosis-4 (aOR, 0.58; 95% CI, 0.40–0.84). The presence of ketonuria in NAFLD patients without prediabetes and DM may have favorable metabolic effects compared to the absence of ketonuria, independent of traditional metabolic factors.

## 1. Introduction

Nonalcoholic fatty liver disease (NAFLD), characterized by hepatic fat infiltration without a secondary cause of fatty liver, is closely associated with diabetes mellitus (DM), obesity, and metabolic syndrome due to insulin resistance (IR) [1,2]. The prevalence of NAFLD is approximately 30% in South Korea, similar to that in Europe and North America (approximately 24%), and is expected to rise due to an increase in the aging population and obesity [2,3,4]. NAFLD represents a wide spectrum of liver diseases with variable prognoses, ranging from simple steatosis, nonalcoholic steatohepatitis, advanced fibrosis, cirrhosis, and hepatocellular carcinoma [1]. It is well known that advanced liver fibrosis and cirrhosis are closely associated with all cause, liver related, and cardiovascular morbidity or mortality [5,6,7,8].

The ketone bodies (KB), including acetone, acetoacetate, and β-hydroxybutyrate (βHB), are derived from the beta oxidation of fatty acids delivered to the liver, and are utilized as an alternative energy source for peripheral tissues, such as the heart, brain, kidneys, and skeletal muscles, upon low glucose availability [9,10,11]. Ketosis, characterized by the increased serum levels of ketone bodies, is divided into pathological and nutritional ketosis [9,12]. Pathological ketosis occurs in uncontrolled DM and hyperglycemia due to insufficient insulin production, and is associated with life threatening conditions, such as diabetic ketoacidosis [12]. However, nutritional ketosis, associated with prolonged fasting and low carbohydrate or ketogenic diets (KD), has been shown to be metabolically favorable in recent studies [13,14,15,16]. In a recent study, KD was shown to have hepatic antisteatotic effects by the activation of mitochondrial beta oxidation in patients with NAFLD [17].

NAFLD has been shown to have a high rate of co-existence with prediabetes and DM [18,19]. Fibrosis and cirrhosis in NAFLD patients were shown in approximately one sixth of prediabetic patients and one fourth of DM patients, respectively [20]. In some reports, the levels of ketone bodies were increased in prediabetic patients and patients with DM [21,22]. However, studies on nutritional ketosis in NAFLD patients without prediabetes and DM are limited. Therefore, we aimed to evaluate the relationship between spontaneous fasting ketonuria and advanced fibrosis in NAFLD patients without prediabetes and DM.

## 2. Materials and Methods

### 2.1. Patients 

The present study was a cross sectional, retrospective study that assessed the association between fasting ketonuria and an intermediate–high probability of advanced liver fibrosis in NAFLD patients without prediabetes and DM. From January 2010 to December 2017, a total of 53,704 individuals who underwent health screening at the Health Promotion Center of Yeungnam University Hospital were identified. Detailed inclusion and exclusion criteria for 10711 NAFLD patients were identified in a previous publication [23].

**Figure 1:**
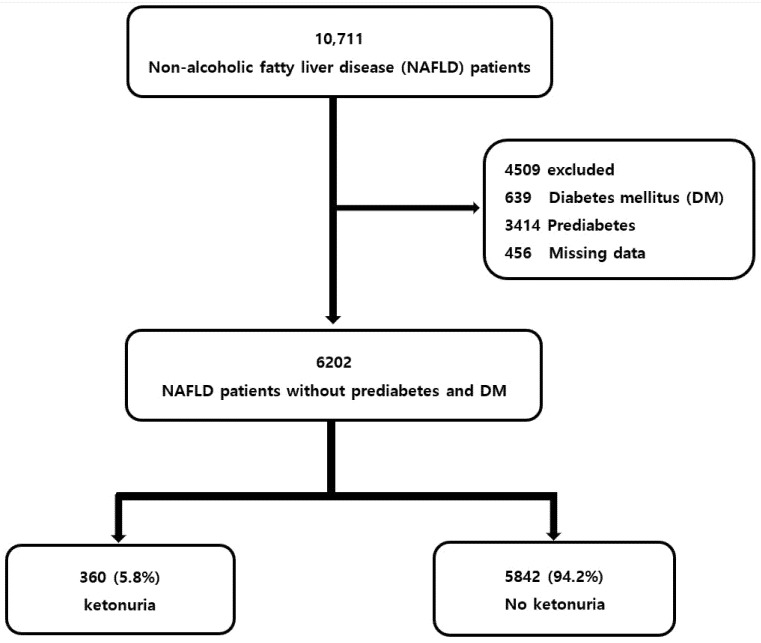
Flow chart of the participants.

Based on the following criteria, 4509 individuals were excluded: (i) patients with DM (*n* = 639), (ii) patients with prediabetes using HbA1c (*n* = 3414), and (iii) inadequate and missing data (*n* = 456). DM was defined as a fasting plasma glucose (FPG) level of ≥126 mg/dL, use of antidiabetic medications, or an HbA1c level of ≥6.5%. In the present study, prediabetes was defined as an HbA1c level of 5.7–6.4% [24]. Meanwhile, 6202 NAFLD patients without prediabetes and DM were included (Figure 1). 

The requirement for informed consent from patients was waived because of the retrospective nature of the study. The study protocol was approved by the institutional review board of Yeungnam University Hospital (IRB No. 2020-03-028).

### 2.2. Assessment of Clinical and Laboratory Variables

Standardized, self-administered questionnaires and anthropometric findings, including height, weight, blood pressure (BP), and waist circumference (WC), were measured. The results of whole blood samples and abdominal ultrasounds (US) were obtained after each patient completed an 8 h overnight fast. The laboratory findings of NAFLD patients, such as serum aspartate aminotransferase (AST), alanine aminotransferase (ALT), gamma-glutamyl transferase (GGT), albumin, platelet count, total cholesterol (TC), high-density lipoprotein cholesterol (HDL-C), low-density lipoprotein cholesterol (LDL-C), triglyceride (TG), FPG, insulin level, high sensitivity C reactive protein (hsCRP), homeostasis model of IR (HOMA-IR), and urinary ketone levels, were measured. 

Using midstream urine specimens, urinary ketone levels were analyzed using semiquantitative urine dipsticks (URiSCAN urine test strips; YD Diagnostics, Yongin, South Korea) within 40 s in a good light, and were classified into five levels: absence, trace (50 mg/L), 1+ (150 mg/L), 2+ (400 mg/L), and 3+ (800 mg/L) based on a color scale. The presence of ketonuria was defined as a level ≥1+ [16]. 

Obesity was defined as a body mass index (BMI) of ≥25 kg/m^2^, based on the Asia-Pacific region criteria [25]. Hypertension was defined as follows: (i) a seated systolic BP of ≥140 mmHg, (ii) a diastolic BP of ≥90 mmHg, and/or (iii) a history of any antihypertensive medication. In the Asian population, metabolic syndrome was defined as the presence of visceral obesity (WC ≥ 90 cm in men and ≥85 cm in women) plus two of the following factors: elevated TG (≥150 mg/dL), reduced HDL-C (≤40 mg/dL in men and ≤50 mg/dL in women), elevated BP (systolic/diastolic BP ≥ 130/85 mmHg), and elevated FPG (≥100 mg/dL), based on the International Diabetes Federation criteria [26]. 

### 2.3. Assessment of Fatty Liver and Probability of Advanced Fibrosis 

Fatty liver was defined by two radiologists using EPIQ 5 and EPIQ 7 (Philips, Amsterdam, The Netherlands) based on the following criteria from our previous publications [23,27]: (i) increased echogenicity of the liver parenchyma relative to that of the cortex of the right kidney, (ii) deep beam attenuation, and (iii) blurring of the intrahepatic vessels [28]. NAFLD was diagnosed as fatty liver via the inclusion and exclusion criteria from previous publications that adopted the Asia–Pacific Working Party on Non-alcoholic Fatty Liver Disease guidelines [23,27,29].

**Figure 2:**
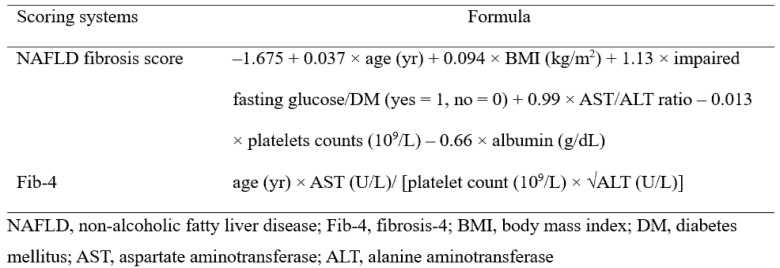
Noninvasive scoring systems for diagnosing of advanced liver fibrosis in patients with nonalcoholic fatty liver disease.

To assess advanced liver fibrosis in NAFLD patients, the NAFLD fibrosis score (NFS) and Fib-4 were used. The two formulae are shown in Figure 2. 

Due to the lack of NAFLD patients with advanced fibrosis using high cut off values (COVs) for NFS and Fib-4 in healthy individuals, low COVs of −1.455 for NFS and 1.30 for Fib-4 were defined as an intermediate–high probability of advanced liver fibrosis in the present study [23,27].

### 2.4. Statistical Analysis

All statistical analyses were performed using R software (version 3.0.2; R Foundation for Statistical Computing, Vienna, Austria). R 3.0.2 was released (26 September 2013) and IBM SPSS version 25.0 (IBM Corp., Armonk, NY, USA). Continuous variables are expressed as mean ± standard deviation or as numbers (%). Differences in variables between the nonketonuria and ketonuria groups in NAFLD patients were calculated using Student’s t-test or chi-squared test. The association between the presence of ketonuria and advanced liver fibrosis using NFS and Fib-4 was identified using logistic regression analysis. With the exception of the variables included in the noninvasive scoring methods for advanced liver fibrosis, we performed sequential adjusted models with confounding variables through multivariate regression analysis. Statistical significance was defined as *p* < 0.05.

## 3. Results

### 3.1. Baseline Characteristics 

Baseline characteristics stratified by the presence of fasting ketonuria are summarized in Table 1. Of the 6202 NAFLD patients without prediabetes and DM, 360 (5.8%) were categorized into the ketonuria group. Compared to the nonketonuria group, the ketonuria group was younger (41.1 ± 10.0 vs. 44.6 ± 11.2 years, *p* < 0.001), had lower levels of FPG (87.2 ± 9.1 vs. 91.0 ± 8.1 mg/dL, *p* < 0.001), insulin (4.1± 2.3 vs. 6.2 ± 3.7 microU/mL, *p* < 0.001), and HOMA-IR (1.0 ± 0.5 vs. 1.5 ± 0.9, *p* < 0.001).

**Figure 3:**
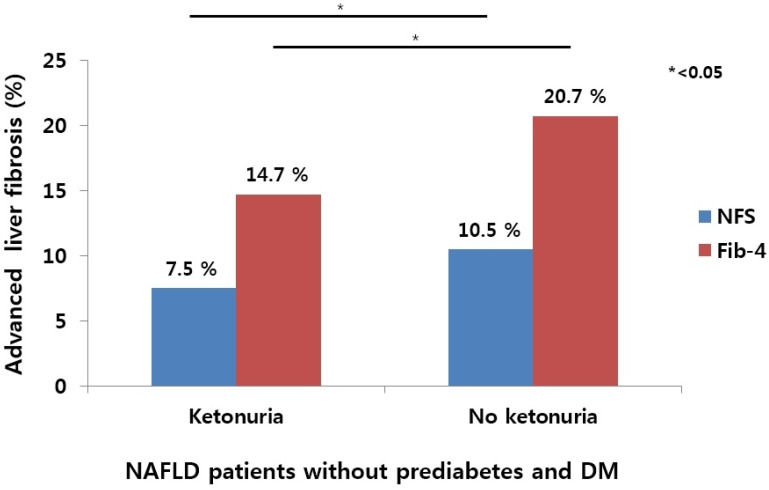
Comparison of percentage of intermediate–high probability of advanced liver fibrosis defined by two noninvasive scoring systems according to presence and absence of fasting ketonuria in NAFLD patients without prediabetes and DM.

The levels of NFS and Fib-4 in the ketonuria group were lower than those in the nonketonuria group (−3.0 ± 1.0 vs. −2.8 ± 1.1 for NFS; 0.9 ± 0.4 vs. 1.0 ± 0.6 for Fib-4, *p* < 0.001). Using low COVs by two fibrosis scoring systems, the ketonuria group had a lower percentage of an intermediate–high probability of advanced liver fibrosis compared to the nonketonuria group (7.5 vs. 10.5% for NFS; 14.7 vs. 20.7% for Fib-4, *p* < 0.05) (Figure 3).

### 3.2. Univariate Analysis for Ketonuria in Patients with NAFLD

On univariate analysis, the factors associated with the presence of fasting ketonuria in NAFLD patients without prediabetes and DM are summarized in Table 2. Lower age (odds ratio [OR], 0.97; 95% confidence interval [CI], 0.96–0.98; *p* < 0.001), high albumin level (OR, 1.89; 95% CI, 1.31–2.72; *p* < 0.001), high LDL-C (OR, 1.03; 95% CI, 1.01–1.06; *p* = 0.017), and high hsCRP (OR, 1.05; 95% CI, 1.02–1.08; *p* = 0.046) were associated with the presence of ketonuria. In addition, lower NFS (OR, 0.83; 95% CI, 0.75–0.91; *p* < 0.001) and Fib-4 (OR, 0.65; 95% CI, 0.50–0.84; *p* < 0.001) were associated with the presence of ketonuria (Table 2). To evaluate the association between the presence of ketonuria and advanced liver fibrosis in NAFLD patients, we performed multivariate adjusted analysis, except for the variables constituting the formulas of NFS and Fib-4. 

### 3.3. Association between Fasting Ketonuria and Intermediate–High Probability of Advanced Liver Fibrosis Defined by NFS 

Adjusted ORs of ketonuria for advanced liver fibrosis, defined by a low COV of NFS, are summarized in Table 3. In the sequential adjusted models, variables for age, BMI, presence of DM, AST, ALT, platelets, and albumin as components of the formula of NFS were not included. The relationship between ketonuria and an intermediate–high probability of advanced liver fibrosis, using NFS, was maintained after adjusting for sex, hypertension, and obesity (Model 1: OR, 0.67; 95% CI, 0.45–1.01; *p* = 0.045); after further adjustment for lipid profiles (Model 2: OR, 0.67; 95% CI, 0.46–1.01; *p* = 0.044); after final adjustment for hsCRP and HOMA-IR (Model 3: OR, 0.67; 95% CI, 0.45–1.01; *p* = 0.044) (Table 3).

The multivariable model was not adjusted for age, BMI level, presence of DM, AST, ALT, platelet, and albumin, which were used to calculate the NFS. 

Model 1: Sex, presence of hypertension, and obesity.

Model 2: Further adjusted for TC, TG, HDL-C, and LDL-C.

Model 3: Further adjusted hsCRP and homeostatic model assessment of IR.

### 3.4. Association between Fasting Ketonuria and Intermediate–High Probability of Advanced Liver Fibrosis Defined by Fibrosis-4

Adjusted ORs of ketonuria for advanced liver fibrosis defined by a low COV of Fib-4 are summarized in Table 4. The adjusted model was not stratified by the components of Fib-4, including age, AST, ALT, and platelet count. The relationship between ketonuria and an intermediate–high probability of advanced liver fibrosis, using NFS, was not attenuated after adjusting for sex, hypertension, and obesity (Model 1: OR, 0.65; 95% CI, 0.48–0.87; *p* = 0.005); after further adjustment for lipid profiles (Model 2: OR, 0.65; 95% CI, 0.48–0.88; *p* = 0.004); after final adjustment for albumin, GGT, hsCRP, and HOMA-IR (Model 3: OR, 0.58; 95% CI, 0.40–0.84; *p* = 0.016) (Table 4).

The multivariable model was not adjusted for age, AST, ALT, and platelet levels, which were used to calculate the Fib-4 index.

Model 1: Sex, presence of hypertension, and obesity 

Model 2: Further adjusted for TC, TG, HDL-C, and LDL-C levels.

Model 3: Further adjusted for albumin, GGT, hsCRP, and homeostatic model assessment-IR 

## 4. Discussion

In the present study, we demonstrated the association between spontaneous fasting ketonuria and an intermediate–high probability of advanced liver fibrosis using low COVs of NFS and Fib-4 in NAFLD patients without prediabetes and DM. Using the stepwise adjustment of two different fibrosis scoring systems, fasting ketonuria was found to be significantly associated with a reduced risk of an intermediate–high probability of advanced liver fibrosis by approximately 30%, independent of traditional metabolic factors. 

Nutritional ketosis in the context of prolonged fasting involves the following steps [17]: First, according to decreased glucose production during fasting, serum insulin concentrations are reduced and hepatic glycogenolysis is accelerated. Second, the process of hydrolysis of TGs in the liver and adipose tissue proceeds. Third, the increased hepatic delivery of fatty acids undergoes mitochondrial β-oxidation to acetyl-CoA, the formation of acetoacetyl-CoA, and conversion to 3-hydroxy-3-methylglutaryl-CoA and acetoacetate [30,31].

However, studies on nutritional ketosis in patients with NAFLD have been poorly characterized. As previously mentioned, NAFLD is closely related to DM and prediabetes because of the common mechanism of IR with compensatory hyperinsulinemia [17,32]. Exacerbation of IR induces excessive glucose production in the liver and hepatic delivery of non-esterified fatty acids (NEFA) in adipose tissue [17]. Excessive NEFA may accelerate the de novo lipogenesis (DNL) of fatty acids, leading to increased intrahepatic TG accumulation [17,33]. 

In our study, although metabolism in NAFLD differed from that of nutritional ketosis, we attempted to show that the group with nutritional ketosis acts may have metabolic superiority compared to that without nutritional ketosis, in patients with NAFLD. Since several studies have reported that KB levels were elevated in prediabetic patients and patients with DM, our study aimed to investigate the association between ketosis and metabolic superiority in NAFLD patients without prediabetes and DM [21,22,34].

To estimate KB levels for diagnostic accuracy, blood KB levels are better than urine KB levels [35]. However, urine KB levels are positively correlated with serum KB concentrations and are considered meaningful biomarkers for estimating hepatic HB production [36]. The presence of ketonuria is related to a higher fat oxidation ability compared to the absence of ketonuria. In the present study, the levels of insulin, HOMA-IR, FPG, and TG were significantly lower in the ketonuria group than in the nonketonuria group. In NAFLD patients without prediabetes and DM, the ketonuria group may have been metabolically favored compared to the nonketonuria group. 

Recently, Kim et al. demonstrated that spontaneous fasting ketonuria was significantly associated with a reduced risk of incident DM over a 12-year prospective study [16]. The incidence rate of DM was approximately 37% lower in patients with spontaneous ketonuria than in those without ketonuria [16]. Kim et al. demonstrated, in a longitudinal study, that fasting ketonuria was associated with a decreased risk of incident NAFLD with/without advanced fibrosis in participants without diabetes [37]. Similarly, the presence of fasting ketonuria had approximately a 30% reduced risk for advanced liver fibrosis in NAFLD patients without prediabetes and DM in our cross sectional study. The differences from the previous study are as follows: First, only patients with pure NAFLD, excluding prediabetes and DM patients, were included in the study. Considering that the levels of ketone bodies were increased in prediabetic patients and patients with DM, our study is significant in identifying fasting ketonuria and fibrosis in pure NAFLD patients, excluding prediabetic patients and patients with DM. Second, the compositions of the adjusted variables are different. Previous studies have suggested an association between fasting ketonuria and fibrosis by adjusting diet and exercise using a multivariate model [37]. In our study, by adjusting the traditional metabolic components of NAFLD, an association between fasting ketonuria and fibrosis was observed regardless of traditional metabolic risk factors. Third, while a previous study revealed a relationship between the development of NAFLD and fibrosis and fasting ketonuria [37], our study differs in that it revealed an association between fibrosis and fasting ketonuria in NAFLD patients at a certain point in time.

Despite being the gold standard tool for the diagnosis of fibrosis, liver biopsy has the following limitations: lethal complications including uncontrolled bleeding and death, sampling bias, and inter and intraobserver variability, and it is an inapplicable method for large cohort studies [38]. Therefore, NFS and Fib-4, which consist of anthropometric and biochemical variables, have been widely used to predict advanced fibrosis in large cohorts with NAFLD [23]. Generally, NFS and Fib-4 have dual COVs, which are the criteria for subdividing the probability of advanced liver fibrosis into low, intermediate, and high. Using the low COVs of the two formulas, the presence of advanced liver fibrosis was defined as an intermediate–high probability of advanced liver fibrosis in this study. 

The association between fasting ketonuria and liver fibrosis in patients with NAFLD is unknown, and the putative mechanism is as follows. First, excess KB production during fasting is strongly correlated with mitochondrial beta-oxidation, which leads to the downregulation of the hepatic DNL pathway and decreased hepatic fat infiltration [17]. In our previous study, abundant visceral adiposity was independently correlated with advanced liver fibrosis in patients with biopsy proven NAFLD. (OR, 6.77; 95% CI, 1.81–29.90; *p* = 0.007) [39]. Considering that KB is associated with fatty acids derived from adipocyte lipolysis during fasting, an inverse relationship between KB production and advanced liver fibrosis may exist in NAFLD patients. 

Second, fibroblast growth factor 21 (FGF21), a liver derived hormone, is activated by fasting or starvation [40]. FGF21 is known to have anti-inflammatory effects and increased mitochondrial beta oxidation by inducing ketogenesis through the peroxisome proliferator activated receptor alpha pathway [33,40]. FGF21 has antifibrotic effects in the liver, which inhibit the activation of hepatic stellate cells [40]. However, abundant proinflammatory cytokines, including nuclear factor kappa B and interleukin-6, exacerbate hepatic inflammation and fibrosis in patients with NAFLD [40,41]. The production of KB in NAFLD patients may be associated with metabolic superiority, including antifibrotic effects, and ketonuria, correlated with serum KB, and might be a favorable metabolic effect. 

Careful interpretation is needed owing to the limitations of the present study. First, because of its cross sectional, single center, retrospective nature, it is difficult to determine the causality between ketonuria and advanced liver fibrosis and to generalize the results to patients with NAFLD. In a recent study, the percentage of spontaneous fasting ketonuria in a nondiabetic population based cohort was 2.2%, which differed from the 5.8% in our NAFLD patient based cohort [16]. Further longitudinal, multicenter, prospective studies are required to confirm the causal association between ketogenesis and liver fibrosis in patients with NAFLD. Second, a direct correlation between ketonemia and ketonuria was not established in this study. However, considering the positive correlation between urine and serum KB production, semiquantitative urine tests may be more cost-effective than blood tests for estimating KB in a large cohort study [36]. Third, the patients’ exercise and dietary information affecting nutritional ketosis were not available in our study. In some studies, diet information affecting nutritional ketosis has a favorable effect in patients with NAFLD. Crabtree et al. demonstrated that hypocaloric low fat and KD may have beneficial effects in reducing fat accumulation in patients with NAFLD [13]. Vilar-Gomez et al. demonstrated that a carbohydrate restricted diet significantly improved the surrogate markers of NAFLD and advanced fibrosis in patients with DM [42]. Luukkonen et al. demonstrated that KD is an effective nutritional intervention for patients with NAFLD [17]. KD activated mitochondrial beta oxidation and the acetyl-CoA pathway, despite the increased hepatic delivery of NEFA and excessive hepatic accumulation of TG due to IR, and compensatory hyperinsulinemia in patients with NAFLD. In addition, KD increased hepatic ketogenesis, including βHB and acetate, and improved plasma glucose, TG, and insulin levels [17]. Considering the positive correlation between urine and serum KB production, fasting ketonuria may be associated with KD. Consequently, KD is closely associated with nutritional ketosis in patients with NAFLD. However, due to the lack of information on the exercise and nutritional survey in our study, it was difficult to determine the physical activity, dietary information including regimens, quantity, and the composition ratio of carbohydrates, proteins, and fats. Future controlled studies, including various dietary regimens and nutritional ketosis in patients with NAFLD, are warranted. Fourth, liver biopsy is essential to elucidate the stage of liver fibrosis. In our study, noninvasive methods using serologic markers were used, instead of liver biopsy, for the diagnosis of advanced fibrosis, which makes it difficult to accurately determine the definite fibrosis stage. In addition, we could not apply other fibrosis assessment tools using transient elastography or shear wave elastography because they were not included in the routine tests of our health promotion center. Although it is difficult to perform a liver biopsy in a retrospective, large population based study, fibrosis assessment tools using transient elastography or shear wave elastography are a challenge to be solved in future studies. Finally, US was used to diagnose fatty liver, which has a subjective interpretation as well as low detection in the presence of mild steatosis (<33%) [28].

## 5. Conclusions

Although NAFLD is associated with IR, the presence of ketonuria in NAFLD patients without prediabetes and DM may have a favorable metabolic effect and reduce advanced liver fibrosis compared to the absence of ketonuria, independent of classic metabolic factors. In NAFLD patients without prediabetes or DM, spontaneous fasting ketonuria may be a novel trademark for estimating the probability of advanced liver fibrosis.

## Figures and Tables

**Table 1:** Baseline characteristics.

Variable	Ketonuria *n* = 360 (5.8%)	No Ketonuria *n* = 5842 (94.2%)	*p*-Value
Age (yr)	41.1 ± 10.0	44.6 ± 11.2	<0.001
Male, *n* (%)	185 (51.4)	2907 (49.8)	0.585
BMI, kg/m^2^	23.3 ± 3.5	23.3 ± 3.1	0.835
WC (cm)	79.6 ± 9.3	79.2 ± 8.5	0.508
Comorbidities			
Obesity, *n* (%)	100 (27.8)	1595 (27.3)	0.892
Hypertension, *n* (%)	32 (8.9)	432 (7.4)	0.346
Metabolic syndrome, *n* (%)	15 (4.2)	329 (5.6)	0.289
Liver profiles			
AST, IU/L	26.0 ± 22.0	23.8 ± 10.7	0.063
ALT, IU/L	27.6 ± 24.1	24.1 ± 19.1	0.008
PLT, K/uL	242.8 ± 53.1	243.3 ± 56.1	0.866
GGT, IU/L	28.2 ± 32.0	27.5 ± 29.1	0.695
Albumin, g/dL	4.8 ± 0.3	4.6 ± 0.3	0.001
Glucose profiles			
FPG, mg/dL	87.2 ± 9.1	91.0 ± 8.1	<0.001
Insulin level, microU/mL	4.1 ± 2.3	6.2 ± 3.7	<0.001
HOMA-IR	1.0 ± 0.5	1.5 ± 0.9	<0.001
Lipid profiles			
TC, mg/dL	198.2 ± 35.3	199.2 ± 34.6	0.616
TG, mg/dL	96.7 ± 64.6	121.2 ± 75.5	<0.001
HDL, mg/dL	59.2 ± 15.7	59.4 ± 15.2	0.775
LDL, mg/dL	119.7 ± 34.3	115.5 ± 32.1	0.117
hsCRP, mg/dL	0.11 ± 0.25	0.09 ± 0.17	0.143
Fibrosis scoring system			
NFS	−3.0 ± 1.0	−2.8 ± 1.1	<0.001
Fib-4	0.9 ± 0.4	1.0 ± 0.6	<0.001

Values are presented as mean ± standard deviation or number (%), unless otherwise specified. BMI, body mass index; WC, waist circumference; AST, aspartate aminotransferase; ALT, alanine aminotransferase; PLT, platelet count; GGT, gamma-glutamyl transferase; FPG, fasting plasma glucose; HOMA-IR, homeostasis model of insulin resistance; TC, total cholesterol; TG, triglyceride; HDL, high density lipoprotein; LDL, low density lipoprotein; hsCRP, high sensitivity C reactive protein; NFS, NAFLD fibrosis score; Fib-4, fibrosis-4.

**Table 2:** Univariate analysis of risk factors for presence of ketonuria in patients with nonalcoholic fatty liver disease.

Variables	OR	95% CI	*p*-Value
Age, years	0.97	0.96–0.98	<0.001
Females	0.94	0.76–1.16	0.549
Obesity	1.02	0.80–1.29	0.844
Hypertension	1.22	0.82–1.75	0.296
Waist circumference, cm	1.00	0.99–1.02	0.470
Aspartate aminotransferase, IU/L	1.01	1.00–1.02	0.003
Alanine aminotransferase, IU/L	1.00	1.00–1.01	0.002
Platelet count, K/µL	1.08	0.74–1.45	0.866
Gamma-glutamyl transferase, IU/L	1.08	0.74–1.45	0.669
Albumin, g/L	1.89	1.31–2.72	<0.001
Total cholesterol, mg/dL	0.99	0.99–1.00	0.616
Triglyceride, mg/dL	1.02	0.75–1.29	0.903
High density lipoprotein, mg/dL	1.00	0.99–1.01	0.775
Low density lipoprotein, mg/dL	1.00	0.99–1.01	0.017
hsCRP, mg/dL	1.00	0.97–1.03	0.046
NFS	0.83	0.75–0.91	<0.001
Fib-4	0.65	0.50–0.84	<0.001

OR, odds ratio; CI, confidence interval; hsCRP, high sensitivity C reactive protein; NFS, NAFLD fibrosis score; Fib-4, fibrosis-4.

**Table 3:** Adjusted odd ratio of ketonuria for intermediate–high probability of advanced liver fibrosis using NAFLD fibrosis score.

	Ketonuria	
	OR (95% CI)	*p*-Value
OR for Advanced Liver Fibrosis		
Unadjusted	0.69 (0.46–1.03)	0.032
Sex adjusted	0.69 (0.46–1.03)	0.039
Model 1	0.67 (0.45–1.01)	0.045
Model 2	0.67 (0.46–1.01)	0.044
Model 3	0.67 (0.45–1.01)	0.044

NAFLD, nonalcoholic fatty liver disease; OR, odds ratio; CI, confidence interval; COV, cut off value.

**Table 4:** Adjusted odd ratio of fasting ketonuria for intermediate-high probability of advanced liver fibrosis using Fibrosis-4.

	Ketonuria	
	OR (95% CI)	*p*-Value
OR for Advanced Liver Fibrosis		
Unadjusted	0.66 (0.49–0.89)	0.006
Sex adjusted	0.66 (0.49–0.89)	0.006
Model 1	0.65 (0.48–0.87)	0.005
Model 2	0.65 (0.48–0.88)	0.004
Model 3	0.58 (0.40–0.84)	0.016

OR, odds ratio; CI, confidence interval; COV, cut off value.

## Data Availability

The data that support the findings of this study are also available from the corresponding author (M.K.) upon reasonable request.
